# Infection Prevention Performance among Hospital Staff during Vaginal Birth: Results from a Criterion-Based Audit at a Zonal Referral Hospital in Tanzania

**DOI:** 10.24248/eahrj.v5i1.649

**Published:** 2021-06-11

**Authors:** Lærke Vinberg Rasmussen, Enna Sengoka, Eusebius Maro, Godfrey Kisigo, Vibeke Rasch, Bjarke Lund Sørensen

**Affiliations:** a Faculty of Health Sciences, University of Southern Denmark; b Kilimanjaro Christian Medical Centre, Tanzania; c Department of Clinical Research, University of Southern Denmark, Denmark; d Department of Obstetrics and Gynaecology, Sjællands Universitets Hospital, Roskilde Sygehusvej

## Abstract

**Background:**

Healthcare associated infections is a global burden and is one of the main causes of maternal and neonatal morbidity and mortality during the time of labour when admitted to the hospital. Healthcare workers' hands are in most cases the vehicle for transmission of microorganisms from patient to patient. Good hand hygiene practices at the bedside are a simple way of reducing healthcare associated infections. The objective was to assess the impact of a criterion-based audit on infection prevention performance and knowledge during vaginal delivery at a hospital in Tanzania. The quantitative findings were discussed with staff to identify barriers and solutions to quality improvement.

**Methods:**

A mixed-method uncontrolled, before and after intervention study by criterion-based audit was performed at the labour ward at Kilimanjaro Christian Medical Centre. Criteria for best practice were established together with key staff based on national and international guidelines. Sixty clean procedures during vaginal birth were observed and assessed by a structured checklist based on the audit criteria. Baseline findings were discussed with staff and an intervention performed including a short training and preparation of alcohol-based hand rub. Hereafter another 60 clean procedures were observed, and performance compared to the care before the intervention. Furthermore, a knowledge test was performed before and after the intervention.

**Results:**

Hand washing increased significantly after a procedure from 46.7% to 80% (RR=1.71 95% CI; 1.27 to 2.31), the use of alcohol-based hand rub before a procedure from 1.7% to 33.3% *p*<*.001*), and the use of alcohol-based hand rub after procedure from 0% to 30% *p*<*.00l*). After the intervention the mean score for the knowledge test increased insignificantly from 59.3% to 65.3%, (mean difference = 6.1%, 95% CI; −4.69 to 16.88).

**Conclusion:**

The criterion-based audit process identified substandard care for infection prevention at the labour ward. An intervention of discussing baseline findings and a short training session and introducing alcohol-based hand rub resulted in improvements on infection prevention performance.

## BACKGROUND

Healthcare Associated Infections (HCAI), also referred to as “nosocomial” and “hospital infections” are infections not present at the time of admission that affect patients in a hospital.^1^ Although the risk of acquiring HCAI is universal, the global burden is unknown. It is estimated that hundreds of millions of people worldwide are afflicted by infections acquired in hospitals.^2,3^ Healthcare associated infections is more prevalent in low- and middle-income countries than in high-income countries, in particular for patients admitted to intensive care and neonates.^1,3^ An estimated 11% of maternal deaths (more than 30,000 per year) and 36% of the neonatal deaths (800,000–900,000 per year) are due to infectious causes.^4,5^ Some of the factors that put women and neonates at risk of infection in healthcare settings are; prolonged and inappropriate use of invasive devices and antibiotics, overcrowded hospitals, poor knowledge on application of basic infection control measures, understaffing and insufficient equipment.^2,6^ The most frequent maternal HCAI are; urinary tract infections, endometritis, chorioamnionitis and infections due to caesarean sections.^7^ Foetuses and newborns are at risk of acquiring HCAI in utero which can lead to preterm labour and spontaneous abortion. Infections can also be acquired, intrapartum as well as postpartum. Conditions during labour and childbirth such as prolonged labour, rupture of membrane, multiple vaginal examines and manual removal of the placenta are further risk factors.^1,2,6,8^ The World Health Organization (WHO) recommends implementing standard precautions, particularly best hand hygiene practices at the bedside and improve staff education and accountability.^2^

A systematic review and network meta-analysis^9^ found improvements in hand hygiene was associated with reductions in HCAI such as *methicillin* resistant *Staphylococcus aureus* infection *(p=.02).* A study from a hospital in Rwanda found increased hand hygiene compliance to be associated with a significant decrease in post-caesarean wound infection (*p*<*.001*).^10^ Tanzania has a maternal mortality ratio of 524 per 100,000 live births.^11^ It is among the countries with the highest maternal mortality in the world, and infections accounts for an estimated 20% of neonatal^12^ and 11% of maternal deaths.^13^ Several studies at Kilimanjaro Christian Medical Centre (KCMC) in the northern part of Tanzania confirm that infections are a major contributor to morbidity and mortality.^14–16^ From 2003 to 2012, it was estimated that 11% of maternal deaths were caused by sepsis. At 14 different departments of KCMC, the prevalence of HCAI was 14.8% on average^14^ and surgical site infections were observed in 19.4% of surgical patients.^15^

Criterion Based Audit (CBA) is a well-established tool for quality assurance with specific focus whereby clinicians can describe and reflect upon their performance compared to agreed criteria for good practice.^17–22^ Compared to other interventions, it is cost-effective and a change in performance is often seen within a short time of implementation. A specific and focused baseline observation is used for a targeted intervention with a following re-evaluation of practice. The objective of this mixed methodology study was to assess the impact of a CBA on Infection Prevention Performance (IPP) by direct structured observations during vaginal birth at KCMC and knowledge of proper IPP. The quantitative findings were discussed with staff to identify barriers to quality improvement.

## METHODS

### Study Area

This study was conducted at KCMC. Kilimanjaro Christian Medical Centre is a zonal referral hospital located in Moshi town, the regional headquarter of Kilimanjaro region in the northern part of Tanzania. The department of Obstetrics and Gynaecology is divided into 3 units; an Obstetric unit with 59 beds, a Gynaecological unit with 52 beds and a labour ward with 4 beds for delivery and 2 operation theatres.^23,24^ In 2016, there were 3,234 births, 42% by caesarean section.

### Study Design and Participants

This study was a mixed-method uncontrolled, before and after intervention study assessing the impact of CBA for IPP during vaginal birth by structured, direct real time observations and a knowledge test within the framework of a CBA. To triangulate the quantitative findings, these findings were discussed with staff, adding qualitative findings to help identify barriers to quality improvement. The study was carried out between February and June 2017.

All midwives, nurses and doctors attending vaginal birth during the observation period were eligible for inclusion and were asked for informed consent. If participation was accepted, they were asked to fill out a short background information questionnaire and a knowledge test by a Key Feature Questionnaire (KFQ). Out of 49 eligible staff members at the labour ward, 22(45%) were included. Of the 22 participants, 1 (4.55%) refused to answer the knowledge test before the intervention and 7 (31.82%) did not answer the knowledge test after the intervention. All participants accepted to be observed during procedure.

### Study Procedures Criterion-Based Audit

**FIGURE 1:**
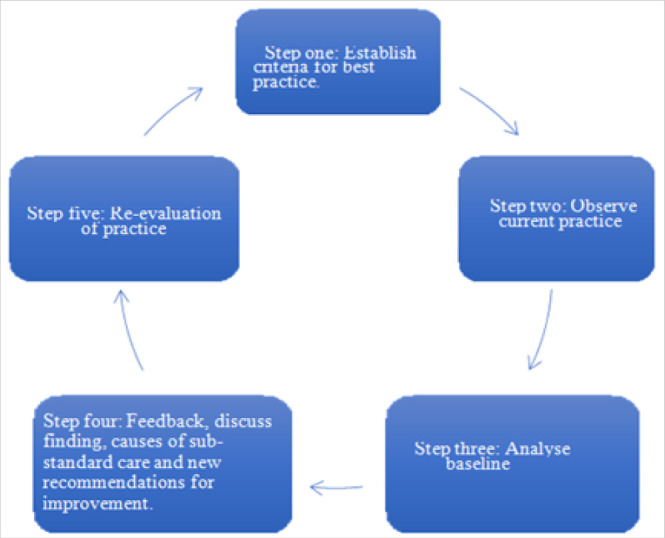
Criterion-Based Audit Presented as a Circle Showing the Steps in the Process

The aim of a CBA is to include staff in reflecting on best practices compared to their actual performance; “like holding up a mirror”^17^ The CBA has 5 steps that are described as audit cycle (Figure 1).

### Step One: Establishing Criteria for Best Practice

In step one, specific audit criteria of key relevance for best realistic practice, considering the resources available, regarding IPP were agreed upon in collaboration with 2 midwives and 2 consultants in obstetrics from KCMC. Criteria were based on national guidelines for hospitals in Tanzania^25^, guidelines by the WHO^2^ and Centers for Disease Control (CDC).^26^

### Step Two: Observe Current Practice

The second step was a 3-week baseline data collection between 1^st^ and 31^st^ of March 2017. The primary outcomes were key procedures for IPP during vaginal labour by a structured checklist including hand wash and the use of Alcohol-Based Hand Rub (ABHR). Secondary outcomes were scores from the KFQ knowledge test. As part of the CBA, staff were discussing barriers to improve quality of IPP and suggestions to overcome them. This qualitative data was included to triangulate the quantitative findings. Observations were un-blinded in real-time using an Objective Structured Observation of Technical Skills (OSATS) developed by the authors based on the audit criteria established in step one. Infection prevention performance was observed in relation to each “clean procedure” defined as vaginal examination, insertion of urinary catheter, insertion of intravenous line, or at birth. Observations were performed at different work-shifts (day, evening and night).

Furthermore, a knowledge test of staff was performed using a validated KFQ developed for the Safe Delivery Application with 7 case-based questions related to IPP.^27^ The structured KFQ for the staff was performed before and after the intervention (step four.)

### Step Three: Analyse Baseline Data

The third step was to analyse the baseline performance as fulfilment rates of each criteria.

### Step Four: Feedback, Discuss Finding and Causes of Sub-Standard Care and New Recommendations for Improvement

Step four was a presentation of baseline findings to the participating staff followed by a discussion of causes of sub-standard care and suggestions for improvements. A short training intervention lasting 2 hours was prepared focusing on the most aggravating substandard care identified at baseline performance. The Safe Delivery application was part of the training.^27^ It is an education aid for skilled birth attendants to improve emergency obstetric care. It has a chapter about Infection Prevention (IP) which was integrated in the session in both English and Kiswahili^28^ with an instruction cartoon video lasting 7 minutes. All participants were invited to the session conducted by the first and second author and notes were taken from the discussion of causes of sub-standard care and suggestions for improvements. Based on the Safe Delivery application, instructions on hand rub was produced by the hospital's pharmaceutical school and distributed at the labour ward ready for use. Posters on how to perform hand hygiene were posted on the wall in the theatre, labour room and triage area.

### Step Five: Re-Evaluations of Practice

The fifth step in the audit circle was a re-evaluation of the practice for another 3 weeks of observations similar to step 2. The findings were compared to the baseline findings. Finally, the results were presented to the staff at the labour ward.

### Statistical Analysis Power Analysis

The global score of IPP was assumed to be 50% at baseline and 75% after the intervention, 59 observations before and after the intervention were thus needed to demonstrate a significant change within a 95% confidence interval with a power of 80. We assumed that on average, 3 observations could be made at each vaginal birth; consequently, 20 vaginal births were to be observed.

The data was entered directly into the statistical software package IBM SPSS 24 (SPSS, Inc., Chicago, IL) and was used for data analysis. Scores for OSATS and KFQ were calculated and presented as percentages of maximum achievable score. All categorical data is presented as fulfilment rates at baseline and after the intervention. Scores were compared by Chi-square test and presented as relative risk scores with 95% Confidence Intervals. At Chi-square test at cells less than 5, a 2-tailed Fisher exact test was used and significance presented by a *p-value* below *.05.* OSATS global scores were compared by Student's t-test, knowledge scores by paired T-test.

### Ethics Approval and Consent to Participate

This study was approved by the ethical review board at KCMC (No. 2025 date of approval 17.02.17). Participants were given verbal and written information about the study. If staff agreed to participate, a consent form was signed. All information was kept anonymous so that poor performance would not influence any individual's employment or relation with superiors.

## RESULTS

The study included 22 out of 49 doctors and midwives at KCMC who were observed during clean procedures before and after the intervention. Their working experience ranged from 2 weeks to 5 years (Table 1). A total of 120 observations were performed, equally divided between before and after the intervention. Two thirds (80 of 120) of the procedures observed were of midwives (Table 2).

**TABLE 1:** Characteristics of Participants

Background data	N = 22 (%)
**Age**	
25–29	10(45.5)
30–34	8(36.4)
≥ 35	1 (4.5)
Missing value	3 (13.6)
**Sex**	
Female	8 (36.4.)
Male	12 (54.5.)
Missing value	2(9.1)
**Time at an obstetrical department**	
<12 Months	11(63.5)
≥ 12	3(13.6)
Months Missing value	8(36.4)
**Profession**	
Midwife/Nurse	7(31.8)
Doctor	13(59.1)
Missing value	2(9.1)

All participants were asked to fill out a short background information questionnaire, but some of the collected questionnaire had missing data, which explains the missing values

**TABLE 2:** Characteristics of Observations among Profession and Specific Procedures

Variable	Frequency n= 120	Percentage n=100%
**Profession**		
Doctors	40	33.3
Midwives	80	66.7
**Name of procedure**		
Vaginal examination	52	43.3
IV catheter	19	15.8
Urine catheter	20	16.7
Vaginal birth	29	24.2

Data are presented in frequency and percentage.

IPP procedures observed showed remarkable substandard care at baseline, some of them improved significantly after the intervention. Hand washing before a procedure was low at 38.3% before increasing insignificantly to 48.3% after the intervention, RR 1.26 (95% CI; 0.83 to 1.91). Hand washing after a procedure increased significantly from 46.7% to 80%, RR 1.71 (95% CI; 1.27 to 2.31). The use of ABHR increased from 1.7% to 33.3% *(p<001*) before a procedure and 0% to 30% *(p<.001*) after a procedure (Table 3).

**TABLE 3:** Direct observations on Infection Prevention Performance

Observation	Baseline n (%) N=60	After intervention n (%) N=60	RR (95% CI) or *p-value*
Hand wash before procedure	23 (38.3)	29 (48.3)	1.26 (0.83–1.91)^*^
Hand wash after procedure	28 (46.7)	48 (80.0)	1.71 (1.27–2.31)
Alcohol-based hand rub before procedure	1 (1.7)	20 (33.3)	p-value 0.00^*^ ^**^
Alcohol-based hand rub after procedure	0 (0)	18 (30.0)	p-value .00^*^ ^**^
Use clean “single-use paper towel” or air-dry	23 (38.3)	22 (36.7)	.96 (.60–1.52)
Wear bracelets/rings/watch	15 (25.0)	23 (38.3)	1.53 (.89–2.64)
Not wearing long sleeves	57 (95.0)	60 (100)	1.05 (.99–1.12)
Not wearing closed footwear	51 (85.0)	40 (66.7)	.78 (.64–.97) ^**^
**At IV catheter**	**N=8**	**N=11**	
Wears non-sterile, clean gloves	6 (75.0)	11 (100)	1.33 (.89–1.99)
**Use of Sterile gloves**	**N=52**	**N=49**	
Wears sterile gloves	52 (100)	49 (100)	
Are sterile gloves applied in the correct way, so sterility is maintained?	32 (61.5)	39 (79.6)	1.29 (1.00–1.67) ^**^
Gloves remains sterile until exploration	39 (75.0)	45 (91.8)	1.22 (1.03–1.46) ^**^
**At sterile procedure**	**N=23**	**N=22**	
Was the instrument sterile when used?	21 (91.3)	22 (100.0)	1.10 (0.97–1.24)
**At cleaning**	**N=21**	**N=18**	
At urine catheter or IV line: Is cleaning performed appropriately?	14 (66.7)	18 (100)	1.50 (1.11–2.03) ^**^

Data are presented in numbers and frequencies and analysed by chi-square test.

* Fishers Exact Test are used when numbers <5.

** Significant result.

**FIGURE 2:**
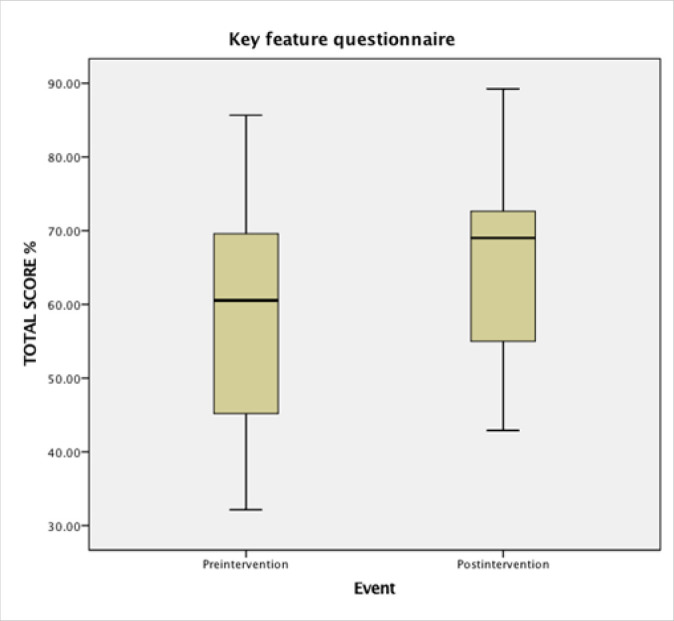
Box and whiskers plot of the results from the Key Feature Questionnaire

15 participants answered the KFQ before and after the intervention. The mean score of their results increased insignificantly from 59.3% to 65.3%, (mean difference = 6.1%, 95% CI; −4.69 to 16.88) (Figure 2). A box and whiskers plot illustrates the results in percentage from the KFQ (Figure 2). Before intervention, the minimum score was =32.1%, Q1=45.2%, median= 60.5%, Q3=69.6 and maximum score was = 85.7%. The interquartile range box represents the middle, 50% of the data and indicate that 50% of the total score is within 45.2% and 69.6%. After intervention, the minimum score was = 42.9%, Q1=55.0%, median=69.0%, Q3=73.6%. In the answers collected after intervention, there are both larger maximum total score and larger minimum total score than before intervention.

15 doctors and midwives participated in the presentation of baseline findings and discussion. Notes were taken from the discussion and divided into 2 main focuses; Suggested reasons for non-compliance of IPP (Table 4) and suggested solutions to overcome non-compliance in IPP (Table 5). Some of the reasons for non-compliance of IPP mentioned by the participants was lack of adequate equipment and lack of accessible hand rub. The staff mentioned that they were convinced that their performance and hand hygiene would improve at the labour ward if hand rub was available (Table 5).

**TABLE 4:** Suggested Reasons for Non-Compliance in Infection Prevention Performance

Discussion issue	Notes from the discussion with staff
Suggested reasons for non-compliance	– Lack of equipment e.g., goggles and aprons. – Not enough lockers in the changing room to lock away values. – Absence of handrub. – Forgetfulness. – Lack of knowledge. – Absence of supervision. – Uncomfortable equipment e.g. goggles that doesn't fit or are difficult to see through during procedures.

**TABLE 5:** Suggested Solutions to Overcome Non-Compliance in Infection Prevention Performance

Discussion issue	Notes from the discussion with staff
Suggested solution to overcome non-compliance	– Wish to be supervised by a nurse specialised in hygiene. – Wish for more knowledge about how to practice IP. – Wish for more supervision. – Wish that a hygiene nurse in charge of IP can go through IP procedures with every new coming staff. – It must be possible to remind each other about IPP. – It should be possible to talk to each other about hand hygiene. – Better changing rooms to be able to lock away values as watches and jewelleries in a safe place. – Hand hygiene would improve if hand rub was available. – Comfortable equipment.

The participants wished to acquire more knowledge regarding correct IPP and wished for a supervisor who could be helpful in situations where standard procedures are forgotten. Due to a high frequency of change of staff in the labour ward, participants suggested that the hospital should employ a hygiene nurse who should orient new staff on IP standards. They also wished to make it legitimate to tell and remind each other about correct IPP.

## DISCUSSION

This CBA identified several fundamentally important IPP to be low at baseline and with improvement after the discussion of findings with staff and a short training session. The knowledge test demonstrated moderate average scores that did not change significantly after the intervention suggesting that knowledge is not necessarily enough to change performance.

The findings for hand hygiene are similar to findings in other studies which indicate that health workers’ hand hygiene in labour wards at baseline is low with ample room for improvement.^10,29–32^ Hand hygiene was observed more often after procedure than before procedure. These results were similar to findings in several other studies.^22^-^29^-^31–33^ A recent review suggests that it is often hand hygiene procedures before patient contact and clean/aseptic procedures, where it is possible to prevent pathogen transmission, that are being neglected.^34^ In a quasi-experimental study at 43 hospitals in 5 low and high-income countries, the study revealed that IP was performed significantly less often before procedures than after. It is discussed as if health providers conceive themselves as “clean” and patients as “unclean”, and that IPP are mainly to protect the health provider not the patient.^29,32^ Another study asked the nurses to give a reason for their behaviour for not washing hands before wearing gloves, they replied back “*it was unnecessary to clean hands because they would be wearing gloves that would prevent microorganism transmission*”.^31^ Whether this is the same belief in the labour ward at KCMC is not possible to say and further studies are needed to shed light on this aspect. Patterns of hand hygiene and behaviour change in healthcare settings are complex therefore studies suggest that multi-level as well as multi-modal strategies are needed.^35,36^

It is mentioned in 2 international guidelines^2,26^ that lack of knowledge on IP has a negative impact on IPP. In this study, a knowledge test was distributed among staff to get an insight on the level of knowledge regarding IP and to identify strengths and weaknesses. The results showed a slightly insignificant improvement after the intervention. Results from a randomised controlled trial study in Indonesia^32^ also investigated the knowledge of the healthcare-staff with the use of WHO's tool.^2^ Their results showed a significant improvement in their knowledge test but concluded that good knowledge cannot alone lead to high hand hygiene compliance.

Alcohol-based hand rub was produced locally at the nearby pharmaceutical school by using WHO-recommended recipes. Local production is recommended as it is at low cost and easy to do and can replace commercial products as well as support local community.^2^ Although ABHR is advised as a cost-effective and effective method for preventing HCAI^2-34-37-38^, it was not accessible for staff at the labour ward. After implementing ABHR in the labour ward, it resulted in a significant increase before and after procedure. A similar increase is found in a pre- and post-intervention study from Rwanda.^10^ Alcohol-based hand rub was introduced at the maternity unit to measure the pre- and post-intervention post-caesarean wound infection rate and hand hygiene compliance.

The intervention of ABHR, involving a half day training session using WHOs tool^2^ and wall mounting visual reminders around ward resulted in a significant increase of hand hygiene compliance for both midwives and doctors from 38.2% to 89.7% as well as a non-significant decrease of post-caesarean wound infections, 6.2% to 2.5%. The authors suggest that the non-significant decrease may be a result of a small sample size studied and short follow-up time.^10^ An uncontrolled before and after interventions study from Tanzania also introduced ABHR at the maternity unit to improve hand hygiene practice during caesarean section. Their findings show that provision of ABHR has contributed to the improvement of hand wash with alcohol hand rub practices especially after the procedure.^22^

The impact of the CBA did not improve the quality of IPP convincingly on all outcomes. One of the factors might be the relatively short period of time compared to other studies that lasted between 16 weeks and 16 months.^10,20,21,30–33^ Another factor could be duration or design of education sessions or the use of a single strategy. A Cochrane review evaluating methods to improve hand hygiene concludes that introducing ABHR, accompanied by education is not enough, multiple strategies are needed.^39^ The observations were carried out during day, evening and night shift over 8-12 hours at a time by the same person, making findings more likely to be representative for the staff at the labour ward. At each procedure, the observer was only observing one staff at a time which decreased the risk for under-recording.

### Strengths and limitations

An uncontrolled study has its limitation as we cannot be sure that the effect we see is due to the intervention and not due to the effect of a confounding variable. Although during the study period we didn’t notice any major changes at the labour ward or any other studies that could have had an effect other than our own intervention. The Hawthorne effect, can lead to overestimation of compliance as staff might improve their performance more as they are partaking in a research project, this is likely to have influenced the study somehow, though the poor baseline performance and only little improvement after the intervention makes it less likely to have had a large influence.^40^

Another limitation is the lack of blinding of the principal investigator. In the second data collection, observations could have been biased towards better outcomes, subconsciously wishing to see positive effects of the intervention. All observations were done without any interruption from the observer making the data comparable and the estimated effect of the intervention reliable. When doing a hypothesis test, there are 2 types of possible errors: Type 1 and Type 2. To avoid Type 1 error, a level of significance was set at 0.05 which mean we were willing to accept a 5% change of being wrong in rejecting the null hypothesis. To avoid Type 2 error, we did a power calculation and ensured that our sample size was large enough.

Compared to other studies, when multiple observers are observing the same phenomena (inter-observer variation), it causes a threat to the variation of observations. This was avoided by using only one observer, who was using the OSATS which made it possible to do structured observations and limit the threat of intra-observer variation.

Another strength was the minimal risk of selection bias. The study had few inclusion criteria as all midwives and doctors were expected to have a basic knowledge of IP through their education and therefore were eligible for participation. Few staff members refused to participate in the study, which might have had an effect on our results toward better or worse outcome.

The CBA is a cost-effective tool for quality improvement in settings with low resources. It permits the staff to learn how often best practice is really followed and by using the CBA compared to other studies, it can help find barriers to good practice. As part of the audit cycle, providing feedback on quality of performance is a way to improve health care workers’ performance.

Criterion based audit in this study, is found to be a useful strategy to improve professional performance in settings with limited resources where baseline of recommended performance is low. The CBA allows the participant to come together and solve the problem as part of the audit cycle. The participants become an important part of formulating practical solutions that improve the quality of care they provide within their setting and thereby finally enriching and improving their professional performance.

## CONCLUSION

In conclusion, the CBA process identified substandard care for IP at the labour ward. An intervention, involving discussion of baseline findings, a short training session and introduction of ABHR resulted in improvements on IPP. Based on the results, it is recommended to Head of Department, health administrators and policy makers that priority is given to the quality of IPP at the labour ward and that CBA might have a place in these efforts as a comparatively cost-effective quality assurance tool that can lead to change relatively fast. However, multi-modal, long lasting strategies are probably more likely to be more effective. It is recommended that ABHR be promoted national wide and to have a specific focus on the availability of ABHR as a simple and feasible intervention to reduce HCAI.
